# Bioprocessing of Wheat and Amaranth Bran for the Reduction of Fructan Levels and Application in 3D-Printed Snacks

**DOI:** 10.3390/foods11111649

**Published:** 2022-06-02

**Authors:** Matea Habuš, Svitlana Mykolenko, Sofija Iveković, Kristian Pastor, Jovana Kojić, Saša Drakula, Duška Ćurić, Dubravka Novotni

**Affiliations:** 1 Faculty of Food Technology and Biotechnology, University of Zagreb, Pierottijeva 6, 10000 Zagreb, Croatia; matea.habus@pbf.unizg.hr (M.H.); sofija.ivekovic@pbf.hr (S.I.); sajredini@pbf.hr (S.D.); dcuric@pbf.hr (D.Ć.); 2 Faculty of Engineering and Technology, Dnipro State Agrarian and Economic University, Serhiy Yefremov 25, 49000 Dnipro, Ukraine; svetlana.mykolenko@gmail.com; 3 BETA Tech Center, TECNIO Network, University of Vic—Central University of Catalonia, C/de Roda 70, 08500 Vic, Spain; 4 Faculty of Technology, University of Novi Sad, Bulevar Cara Lazara 1, 21000 Novi Sad, Serbia; pastor@tf.uns.ac.rs; 5 Institute of Food Technology, University of Novi Sad, Bulevar Cara Lazara 1, 21000 Novi Sad, Serbia; jovana.kojic@fins.uns.ac.rs

**Keywords:** bran fermentation, FODMAP, fructose, lactic acid bacteria, *Kluyveromyces marxianus*, *Saccharomyces cerevisiae*, inulinase, 3D-printability

## Abstract

Bran can enrich snacks with dietary fibre but contains fructans that trigger symptoms in people with irritable bowel syndrome (IBS). This study aimed to investigate the bioprocessing of wheat and amaranth bran for degrading fructans and its application (at 20% flour-based) in 3D-printed snacks. Bran was bioprocessed with *Saccharomyces cerevisiae* alone or combined with inulinase, *Kluyveromyces marxianus*, *Limosilactobacillus fermentum,* or commercial starter LV1 for 24 h. Fructans, fructose, glucose, and mannitol in the bran were analysed enzymatically. Dough rheology, snack printing precision, shrinkage in baking, texture, colour, and sensory attributes were determined. The fructan content of wheat bran was 2.64% dry weight, and in amaranth bran, it was 0.96% dry weight. Bioprocessing reduced fructan content (up to 93%) depending on the bran type and bioprocessing agent, while fructose and mannitol remained below the cut-off value for IBS patients. Bran bioprocessing increased the complex viscosity and yield stress of dough (by up to 43 and 183%, respectively) in addition to printing precision (by up to 13%), while it lessened shrinkage in baking (by 20–69%) and the hardness of the snacks (by 20%). The intensity of snack sensory attributes depended on the bran type and bioprocessing agent, but the liking (“neither like nor dislike”) was similar between samples. In conclusion, snacks can be enriched with fibre while remaining low in fructans by applying bioprocessed wheat or amaranth bran and 3D printing.

## 1. Introduction

Fermentable oligo-, di-, and monosaccharides and polyols (FODMAPs) are not digestible in the human small intestine but are rapidly fermented by the gut microbiota. FODMAPs include fructans, galacto-oligosaccharides (GOS), lactose, fructose in excess to glucose, sorbitol, mannitol, and xylitol [1,2,3]. Fructans are considered prebiotics for healthy people and are an important source of sugar for yeast fermentation in bread making [4]. Nevertheless, they can trigger abdominal pain, swelling, constipation, and diarrhoea in patients with irritable bowel syndrome (IBS) and non-celiac gluten sensitivity (NCWS) [2,4,5]. For these patients, it is necessary to reduce the total intake of FODMAPs below the cut-off value of 0.5 g per serving [1,3]. At the same time, eliminating foods rich in fructans could result in insufficient intake of dietary fibre and micronutrients as well as undesirable changes in the gut microbiota [4,6]. The market for low FODMAPs products is based on gluten-free products that are not sensory attractive and have low nutritional value [7]. So, there is a need to develop high-fibre but low-FODMAPs food.

Whole grain wheat, rye, and barley contain a high amount of dietary fibre, but also fructans and GOS [1,8,9], which are mainly concentrated in the bran [4,8,10,11]. Gluten-free grains such as oats, millet, rice, and maise contain small amounts of FODMAPs [9,12]. Although gluten-free, some pseudo-cereals such as amaranth have a dubious reputation for containing FODMAPs. Processed products made from amaranth grains, which are rich in micronutrients and bioactive compounds, can have high FODMAPs levels [13]. Still, the FODMAPs content of amaranth bran (AB) has not been reported yet.

Hydrolysis of cereal fructans and GOS can occur by germination, fermentation with microbial cultures, or with various enzymes, such as *α*-galactosidase, inulinase, or invertase [2,3,5,14]. Enzymatic degradation of fructans has already been achieved in whole wheat flour and lentils [1] as well as in agave juice [15]. For the degradation of wheat FODMAPs with fermentation, the activity of fructanase and invertase was proved to be crucial [14,16,17]. Bakery yeast (*Saccharomyces cerevisiae*) produces the enzyme invertase [3,4,18], while *Kluyveromyces marxianus* produces the enzyme fructanase [17,19]. They are often used as co-cultures in the production of whole wheat bread [4,5,11,17,19,20], rye bread, and chapatti [5,6] as *K. marxianus* lacks the ability to degrade maltose, the main fermentable sugar produced in starch hydrolysis [3,19]. In addition to the degradation of fructans, the *S. cerevisiae*-derived invertase also catalyses the hydrolysis of sucrose and raffinose oligosaccharides [4]. Longer fermentation with lactic acid bacteria could lead to more successful degradation of FODMAPs in bran [2,3,5]. The use of different *Lactobacillus* species results in fructan degradation in wheat steamed bread [2] and wheat bran (WB) [21] and has been suggested to reduce fructan content in malt [7]. However, some lactic acid bacteria, such as *Levilactobacillus brevis* or *Leuconostoc citreum,* are able to convert sugars into mannitol [22]. According to our knowledge, no study has investigated the effect of inulinase, *S. cerevisiae*, *K. marxianus,* or *L. fermentum* on the removal of fructan from wheat or amaranth bran.

Wholegrain products tailored to specific dietary needs, e.g., with reduced FODMAPs content, could be offered as healthy snacks. Healthy snacks are a rising popular food category, as consumers seek low-sugar and salt and high fibre snacks [23]. Three-dimensional (3D) extrusion-based printing represents a novel approach for producing nutritionally adapted and balanced cereal snacks [24,25]. It provides a possibility of using alternative ingredients, but it is necessary to understand their rheological properties, i.e., their ability to flow and support the given 3D structure [25,26,27], as well as the ability of the dough to resist deformation during post-processing [28]. In recent years, several studies reported success in the 3D printing of snacks [24,26,29]. To our knowledge, no study has investigated the impact of fructans removal techniques or fermentation in general on the 3D-printability of snacks. Thus, the aim of this study was to investigate the bioprocessing of WB and AB for the removal of fructans and their application in 3D-printed snacks. Bran was bioprocessed with bakery yeast *Saccharomyces cerevisiae* (BY) alone or combined with enzyme inulinase, yeast *Kluyveromyces marxianus*, or lactic acid bacteria (LAB) *Limosilactobacillus fermentum* or using a commercial starter of mixed yeast and LAB cultures.

## 2. Materials and Methods

### 2.1. Materials

WB was a gift from an industrial Farina mill (Granolio Inc., Zagreb, Croatia), while AB was kindly provided by RICHOIL (Lviv, Ukraine) and the Association of Amaranth Producers and Processors (Dnipro, Ukraine). WB contained 17.8% protein, 12.3% moisture, 25.6% carbohydrate, 36.1% dietary fibre (of which soluble 4.6%), 4.3% fat, and 3.6% ash [30]. AB consisted of 17.0% protein, 7.6% moisture, 57.5% carbohydrate, 11.4% fibre (of which soluble 1.7%), 4.2% fat, and 2.3% ash. WB and AB showed unimodal particle size distribution with a median 50th percentile diameter of 177.00 ± 2.26 and 242.08 ± 0.46 µm, respectively, determined using the Mastersizer 2000 instrument equipped with the Scirocco 2000 dry dispersion unit (Malvern Instruments, Worcestershire, UK) [30]. Livendo^TM^ LV1 starter, containing *Levilactobacillus brevis*, *Lacticaseibacillus casei*, and *Saccharomyces chevalieri*, was donated by Lesaffre Adriatic Inc. (Prigorje Brdovečko, Croatia). *Limosilactobacillus* *fermentum* (DSM 20052) was provided by Deutsche Sammlung von Mikroorganismen and Zellkulturen (DSMZ, Braunschweig, Germany), while *Kluyveromyces marxianus* (NBRC 1777) was donated by the Laboratory for Biochemical Engineering, Industrial Microbiology and Malting and Brewing Technology, Faculty of Food Technology and Biotechnology University of Zagreb. Inulinase from *Aspergillus niger* (EC 3.2.1.26., 2000 U/g) was kindly provided by BIO-CAT (Troy, VA, USA). Dry BY (Lesaffre Adriatic Inc., Prigorje Brdovečko, Croatia), as well as the ingredients for the dough preparation (oat flour (9.5% proteins, 5.6% lipids, 2.15% fibre (Garden Ltd., Zagreb, Croatia)), rice proteins (83% proteins, 4.5% lipids (Biovega, Ltd., Zagreb, Croatia)), sunflower oil (Zvijezda Ltd., Zagreb, Croatia), salt (Solana Pag Inc., Pag, Croatia), and baking powder (Podravka Inc., Koprivnica, Croatia) were purchased at the local market.

### 2.2. Bioprocessing of Bran

**Figure 1:**
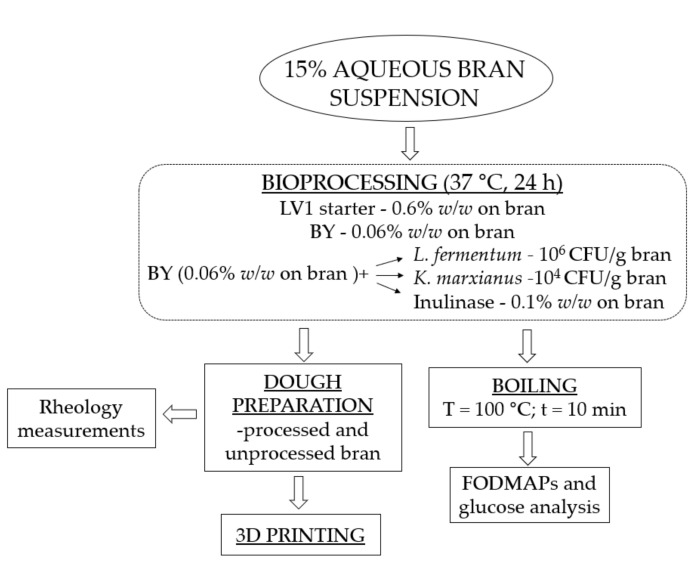
Schematic representation of bran bioprocessing and its use in dough preparation. BY, bakery yeast.

A schematic representation of the experiments is shown in Figure 1. Unprocessed bran served as a control sample. Aqueous suspensions (15% *w*/*w*) of WB or AB were incubated with LV1 starter (0.6% *w*/*w* on bran) or BY *S. cerevisiae* (10^4^ CFU/g or approx. 0.06% *w*/*w* on bran) alone or in co-culture with the following cultures: *K. marxianus* (10^4^ CFU/g bran), *L. fermentum* (10^6^ CFU/g bran), or inulinase (0.1% *w*/*w* on bran) at 37 °C for 24h. Bioprocessing time was defined in preliminary experiments. The pre-culture of *L. fermentum* was prepared in MRS broth (Biolife, Monza, Italy) containing 2% (*w*/*v*) glucose, while the broth medium for the propagation of *K. marxianus* contained yeast extract, peptone, and glucose (1, 2 and 2% *w*/*v*, respectively). Both pre-cultures were incubated at 37 °C. After centrifugation, the cultures were dissolved in sterile water. The inoculum was homogenized by vortexing for 1 min and immediately used for fermentation together with BY. Water addition was subtracted by the amount of water previously added with inoculum.

### 2.3. FODMAP Content

The content of fructans, fructose, glucose, and mannitol before and after bran bioprocessing was analysed enzymatically according to AOAC method 999.03 (with Fructan Assay Kit, Megazyme, Ireland), AOAC method 985.09 (with D-Fructose/D-Glucose Assay Kit, Megazyme, Ireland), and D-Mannitol Assay Kit (Megazyme, Ireland), respectively. Samples for the determination of fructose, glucose, and mannitol were freshly prepared on the day of analysis. Each sample suspension was heated at 80 °C for 10 min in a water bath. After cooling to room temperature and centrifugation at 4000 rpm for 10 min, 1.5 mL of the supernatant was centrifuged again at 14,700 rpm for 3 min [21]. The results for fructans represent the combined content of fructans and galactooligosaccharides, as the samples were not treated with *α*-galactosidase before analysis. A proximate total FODMAP content in 30 g of snack was calculated based on fructans, fructose, and mannitol content determined in the bran (3 g), oat flour (15 g), and rice protein (5 g) used for the preparation of the dough (Section 2.4.), considering the loss of water during baking (i.e., the baking loss). Baking loss was determined using the following formula [24]:

Baking loss (%) = (m_pd_ − m_bs_/m_pd_) × 100 (1)

where m_pd_ represents the weight of dough before baking and m_bs_ the weight of baked snack.

### 2.4. Preparation of Dough

According to our previous study [24], the dough was prepared in three steps but with slight modifications. First, rice proteins, salt, baking powder, and sunflower oil were mixed with a hand mixer (M350LBW, Gorenje, Slovenia) (3 min at low speed), and then the suspension of unprocessed or bioprocessed WB or AB was added (mixing for 1 min at low and 1 min at medium speed), followed by the addition of oat flour (mixing for 2 min at low speed). The dough was immediately used for rheology analysis and 3D printing.

### 2.5. Rheological Properties

All oscillatory measurements were performed using a parallel plate geometry of 25 mm diameter with a 1 mm gap with an MCR 92 rheometer (Anton Paar, Graz, Austria). The amplitude sweep test (with the shear rate of 0.01–100 s^−1^ at a constant frequency of 1 Hz) was performed to determine the linear viscoelastic region (LVER) and the shear strain (0.05%) for the frequency sweep test, which was then employed in the range of 1–30 Hz at 20 °C [24]. Before each test, the dough was placed under the parallel plate and, after putting the plate down, trimmed if necessary. After duplicate measurements, the storage modulus (G′), loss modulus (G″), loss factor (tan δ = G″/G′), yield stress, flow point, and complex viscosity were calculated by the software Anton Paar RheoCompass (version 1.30.999, Graz, Austria).

### 2.6. Three-dimensional Printing and Post-Process Baking

The spiral shape (with 25 layers and a layer height of 0.4 mm) of the dough samples was 3D extruded using Createbot 3D Food Printer-Multi-Ingredient Support (Ningbo Createbot Electronic Technology Co., Ltd., Ningbo, China) and Cura 15.02.1 software. Printing was performed with a nozzle diameter of 1 mm, at a temperature of 20 °C, with a printing speed of 25 mm/s; thus, 407 s was needed to 3D print one sample.

Three-dimensional-printed samples were baked in a deck oven (EBO 64-320 IS 600, Wiesheu GmbH, Germany) for 18 min with the lower heater set at 140 °C and the upper heater at 160 °C [24]. Samples were cooled to room temperature before further analysis.

### 2.7. Three-dimensional Printability and Physical Properties of Baked Snacks

**Figure 2:**
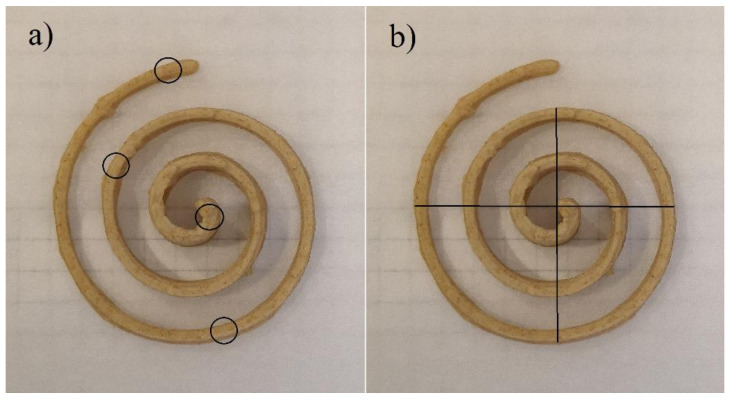
Measuring positions of: (**a**) snack height and line width; (**b**) diameter of baked snacks.

All physical properties were measured in 10 replicates, and the results are provided as mean values. The total height and line width at the top of the baked snack were determined at 4 positions (Figure 2a), while the snack diameter was determined at 2 positions (Figure 2b) using a calliper.

All samples were scanned at 600 dpi (CanoScan, LIDE 2020, Canon, Tokyo, Japan) after 3D printing and baking. The shape accuracy (%) of baked snacks was determined with digital image analysis (ImageJ, National Institutes of Health, Bethesda, MD, USA) as the proportion of black pixels, calculating the deviation of each printed sample from the one printed with the highest precision. In addition to the shape accuracy, printing quality was defined with 3D printing precision and shape shrinkage in baking, which was calculated as demonstrated previously [24]:

Printing precision (%) = (D_L_/D_n_) × 100 (2)

where D_L_ is the width at the top of the 3D-printed dough (cm), and D_n_ is the diameter of the printing nozzle, both in cm.

Shape shrinkage = ( (X_d_ − X_s_ ) ⁄ X_d_) × 100 (3)

where X_d_ and X_s_ are the total white pixels of the 3D-printed dough and baked snack, respectively.

The colour of the baked snacks, i.e., the lightness (L*), redness (a*), and yellowness (b*), was measured with a colourimeter (Konica Minolta CM-700d, Tokyo, Japan). The browning index (BI) for each snack after baking was calculated using the following equation [24]:

BI = 100 × ((((a* + 1.75 × L*)/(5.645 × L* + a* − 3.012 × b*)) − 0.31)/0.17) (4)

The total colour change (ΔE* value) between the first and last 3D-printed and baked snack was calculated [24]:

(5) $$\Delta E*=\sqrt{\Delta {L*}^{2}+\Delta {a*}^{2}+\Delta {b*}^{2}}$$

The hardness of baked snacks was analysed with a cutting test performed at a speed of 2 mm/s [29] using a texture analyser (Ametek Lloyd Instruments Ltd., West Sussex, UK) equipped with a 50 kg load cell and the Warner–Bratzler shear blade guillotine probe.

### 2.8. Sensory Analysis

The sensory evaluation of snacks with unprocessed and bioprocessed bran was carried out by a 13-member panel of semi-trained judges (average age of 35 years comprising 11 female and 2 male), employees from the Faculty of Food Technology and Biotechnology. Panellists had neither food allergies nor intolerances and were informed about the objectives of the study. Samples were randomly coded with three-digit numbers and served in random order in two sessions (containing either WB or AB) on two separate days. The descriptive sensory analysis included attributes related to the uniformity of surface colour, odour (bran, yeast), and taste/flavour (salty, bitter, oil, fermented) evaluated on a scale from not detectable (0) to attribute strongly expressed (5) [31]. Control samples with unprocessed bran were served as reference products with defined intensities of each attribute. A 5-point hedonic scale ranging from extremely dislike (1) to extremely like (5), where 2 = do not like it moderately, 3 = neither like it nor do not like it, and 4 = like it moderately, was used to assess the liking of snacks. Panellists were instructed to clean their palate between the samples with spring water.

### 2.9. Statistical Analysis

Factorial analysis of variance (ANOVA) was performed to establish the influence of the bran type and bioprocessing agents on the FODMAPs level of the bran, rheological properties, and 3D printability of the dough as well as physical parameters of the baked snacks. ANOVA and Tukey’s post hoc test (*p* < 0.05) were carried out with Statistica 10 software (Stat Soft Inc., Tulsa, OK, USA). Principal component analysis (PCA) was performed using a PAST 4.09 software. The data matrix constructed of measured parameters was employed in unsupervised multivariate data processing in order to check the relationships between the investigated variables and 3D-printed snack samples [32].

## 3. Results and Discussion

### 3.1. FODMAP Content

Low fructan content was found in oat flour (0.15% dry weight), and rice proteins (0.14% dry weight) were used to prepare the dough. The fructan content of our unprocessed WB (Table 1) agrees with the previously reported fructans content of 2% (dry weight) in WB [8]. Unprocessed AB contained 2.5-fold fewer fructans compared to WB. The WB and AB were subjected to five different treatments to reduce their fructan content. All treatments resulted in a significantly lower content of fructans and GOS, depending on the interaction between bran type and bioprocessing agent (*p* < 0.01). Fermentation of WB with BY alone resulted in a 63% lower fructans content, while its combination with *L. fermentum* or *K. marxianus* resulted in a greater reduction in fructans and GOS, by 83 and 88%, respectively. In line with this, previous studies showed that the combined action of *S. cerevisiae* and *K. marxianus* leads to a 90% reduction in the fructans content of whole wheat bread, while treatment with bakery yeast alone leads to a 56–80% reduction, depending on BY concentration and fermentation time [3,4,17]. Lower degradation of wheat fructans by BY without *K. marxianus* may be due to a lower specificity of the invertase for higher polymerization oligosaccharides (fructans) as substrates or to the lower activity and amount of the synthesised enzyme from BY compared to the enzyme from *K. marxianus*. The most successful fermentation of WB was with the LV1 starter or the combination of BY with inulinase, both of which led to a 93% in fructans and GOS content. After the fermentation of AB, the fructan and GOS content were very low. Similar to WB, the greatest reduction of fructans and GOS by 92 and 95%, respectively, was also achieved in AB with BY alone or in combination with inulinase. Previously, the reduction of fructans content in WB after 18 h of incubation with different species of lactic acid bacteria ranged from 77% to almost complete degradation (99% reduction when using *Lactobacillus sanfranciscensis*) [21]. In addition, Atzler et al. [1] reported that fructans are not detectable after 2 h incubation of wholemeal wheat flour with inulinase (300 U/mL). Our study shows that fructans can be degraded after long fermentation using yeast only, but in case of high concentrations such as in WB, bioprocessing means should be combined for bigger success.

The hydrolysis of fructans releases glucose and fructose. When present in excess of glucose and exceeding the cut-off value of 0.5 g/100 g [17], fructose is also classified as a FODMAP [2,5]. Untreated, WB and AB contained fructose in a smaller amount compared to glucose (Table 1). Nevertheless, the fructose content increased during the fermentation of WB and even exceeded the glucose content after fermentation with BY in combination with *L. fermentum* or *K. marxianus*. After the 24h fermentation of AB, the fructose content increased only with BY. Co-culture of BY and *K. marxianus* resulted in 11 and 29% higher glucose content compared to the untreated WB and AB, respectively. Fermentation of AB with *S. cerevisiae* alone or in co-culture with *L. fermentum* resulted in 13% higher glucose content. Although fructose and glucose are released during the fermentation, their levels usually remain low as these sugars are consumed by yeasts and lactobacilli [17]. Heterofermentative lactic acid bacteria can further convert the released fructose into mannitol, which is also defined as FODMAP with the recommended cut-off value of 0.2 g per serving [3,14]. Here, mannitol was produced in each bran fermentation (Table 1) in an amount depending on the interaction between bran type and bioprocessing agent (*p* < 0.01). In both WB and AB, bioprocessing with BY and inulinase resulted in the highest mannitol contents. Therefore, it is necessary to follow the degradation products of fructans as they add up to the FODMAP content. However, considering the bran content in the snacks and the average baking loss (45%), the proximate total FODMAP content in 30 g of each snack would remain below the limit (Table 1).

### 3.2. Effect of Bran Bioprocessing on the Rheology and 3D-Printability of the Dough

**Figure 3:**
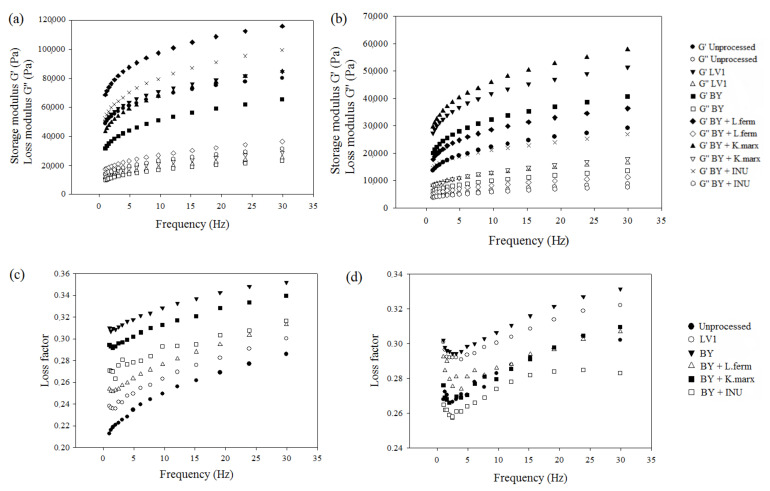
Storage and loss moduli of: (**a**) wheat bran; (**b**) amaranth bran, and loss factor of: (**c**) wheat bran; (**d**) amaranth bran. BY, bakery yeast; INU, inulinase.

The rheological properties investigated in this study were significantly influenced by the interaction between bran type and bioprocessing agent (*p* < 0.01). Higher G′ than G″ values for all samples (Figure 3a,b) indicated their solid-like behaviour, which is crucial for successful 3D printing, i.e., achieving dimensional stability after extrusion-based 3D printing [25]. Both the G′ and G″ of our oat-based dough were up to nine-fold higher compared to wholegrain rye dough with the milk powder, previously reported by Lille et al. [29]. Samples with either WB or AB bioprocessed with BY showed the highest loss factor (Figure 3c,d). This indicated that dough with added bran was bioprocessed with BY demonstrated the most viscoelastic properties, i.e., had the highest ability to absorb energy and relieve stress.

The determined complex viscosity, yield stress, and flow point of the dough with added unprocessed WB (Table 2) did not significantly differ from those of the dough with added pea protein used in our previous study [24]. Moreover, the doughs with the addition of unprocessed WB showed higher complex viscosity, yield stress, and flow point compared to AB. This could be explained by the higher content of fibre, particularly soluble fibre, as well as the difference in fibre composition, i.e., arabinoxylans are highly present in WB [33]. The complex viscosity of the dough containing WB decreased by 9% only after fermentation with the co-culture of BY and *K. marxianus*, while BY alone and co-cultured with *L. fermentum* resulted in significantly higher complex viscosity. Furthermore, the complex viscosity of the dough with AB increased in all treatments, but significant were only bioprocessing with LV1, BY, and its co-culture with *K. marxianus*. AB contain pectin fibre as well as xyloglucans and galacturonans (galacturonic acid and galactose) [34], while *K. marxianus* possesses β-galactosidase, pectinase, and β-xylosidase [35], whose action at acidic pH (ranging between 4.2 and 4.3 in our samples at the end of fermentation) could lead to the solubilization of AB fibre and consequently increase dough viscosity. The addition of fermented WB increased the yield stress of the dough, with the co-culture of BY and L. *fermentum* being the most favourable (Table 2). A similar pattern was observed for AB-containing doughs, except that fermentation with BY and *L. fermentum* resulted in lower yield stress compared to doughs with unprocessed AB. Higher yield stress means that the dough has the ability to form self-supporting layers [36]. Similar to our results, Lille et al. [29] reported yield stress for rye dough ranging from 10 to 58 Pa. Regardless of the bran type, a higher flow point was observed in all bioprocessed bran-containing doughs, indicating that more extrusion should be applied for the dough to begin to flow, which could also be linked with increased swelling and fibre solubility. Previously, the fermentation of amaranth flour [37,38], as well as WB [39,40] with various species of *Lactobacillus,* was found to improve the rheological properties of the dough and the quality of wheat composite bread. Additionally, oat flour provides improved viscosity compared to wheat flour [41]. Our study showed that the fermentation of both WB and AB with only BY or mixed cultures contributes to the dough rheology, unlike its combination with inulinase. Even though the rheology of dough with AB remains inferior to WB-containing dough. This could be due to the fact that WB contained more fibre than AB and additionally contains gluten proteins.

The rheological properties of the dough have the greatest influence on 3D printing precision, which is equivalent to printing quality, as it is highly related to the reproducibility and consistency of 3D printing and the quality of the final product. The printing precision of dough containing unprocessed WB or AB was similarly satisfactory. Fibre-rich ingredients used for dough preparation have already been associated with high printing performance [24,27]. For a high printing precision, the dough needs to possess an appropriate viscosity, i.e., to be easily extruded while supporting the following deposited layers [42]. Indeed, we found a positive correlation (r = 0.73; *p* < 0.01) between the printing precision and the complex viscosity of our samples (Table 2). Bran bioprocessing had a positive or negligible effect on printing precision (Table 2), depending on the interaction between bran type and bioprocessing agent (*p* < 0.01). Dough containing WB fermented with BY or its co-cultures with *L. fermentum*, as well as dough with added AB fermented with BY and *K. marxianus*, were printed the most accurately. Thus, bran bioprocessing can contribute to dough printability, but the bioprocessing agent should be selected depending on the bran type.

### 3.3. Physical and Sensorial Attributes of Snack

No significant difference was found in average weight (1.1 ± 0.0 g), height (4.6 ± 0.1 mm), line width (1.6 ± 0.0 mm), or diameter (35.9 ± 1.6 mm) between the baked snacks as a function of bran nor bioprocessing type (data not shown). The dough was shrunk during baking depending on the bran type and bioprocessing agent (Table 3). On average, dough containing WB was less shrunk than dough with AB. Further on, samples with bioprocessed bran were significantly less shrunk by 20–69% compared to dough with unprocessed bran. This could be related to the difference in dough rheology since an inverse correlation was found between shape shrinkage and dough flow point (r = −0.65, *p* = 0.02). Bioprocessing of both WB and AB with co-culture of BY and *K. marxianus* resulted in the lowest snack shrinkage, respectively. This behaviour might be linked with possibly the highest CO_2_ production rate in the synergistic action of BY and *K. marxianus,* resulting in dough expansion [17].

It is known that bran has high polyphenol oxidase activity, causing undesirable browning during 3D printing due to the slowness of the process [24,30]. In this study, ∆E* between the first and the last (10th) 3D-printed and baked snacks ranged from 0.12 to 0.87, which is defined as a difference in colour barely visible to the human eye [42]. Compared to matching WB, the addition of both unprocessed and bioprocessed AB resulted in significantly lighter, more yellow and less red snacks with a lower browning index (BI) (Table 3). BI is related to Maillard’s reactions, the dextrinisation of starch, and the caramelisation of sugar during baking [42]. In this study, the BI of the snacks was significantly influenced by the interaction between bran and the type of the bioprocessing agent (*p* < 0.01). Compared to samples with unfermented bran, all WB and AB-containing snacks had a higher BI, except those fermented with BY and *L. fermentum*. The higher BI of snacks with WB bioprocessed with BY might be related to the bioavailability of amino acids during fermentation with BY [43], which are known to be involved in the formation of brown pigments [42]. In previous studies, acidic doughs were found to have higher L* and b* values compared to dough with added sodium bicarbonate, but there was no general rule for BI. A similar BI of around 42 was previously observed for 3D-printed snacks with added pre-processed WB [24].

The hardness of snacks was also significantly dependent on the interaction between bran and the type of pre-processing agent (*p* < 0.01). Snacks with WB were harder than snacks containing amaranth bran. The hardness of the snack was significantly correlated with the dough complex viscosity (r = 0.748; *p* = 0.005). Fermentation of WB and AB bran resulted in a lowering of snack hardness, except when WB was fermented with BY and *L. fermentum* (Table 3). Lille et al. [29] reported similar results for the hardness (11–20 N) of 3D-printed snacks as ours. Rani et al. [43] reported that rice-black gram snacks extruded after fermentation with BY had hardness in the range of 15–37 N, depending on barrel temperature, extruder screw speed, and die opening diameter. To the best of our knowledge, there are no studies that have investigated the effects of the bioprocessing agents used in this study on the textural properties of the snacks.

**Figure 4:**
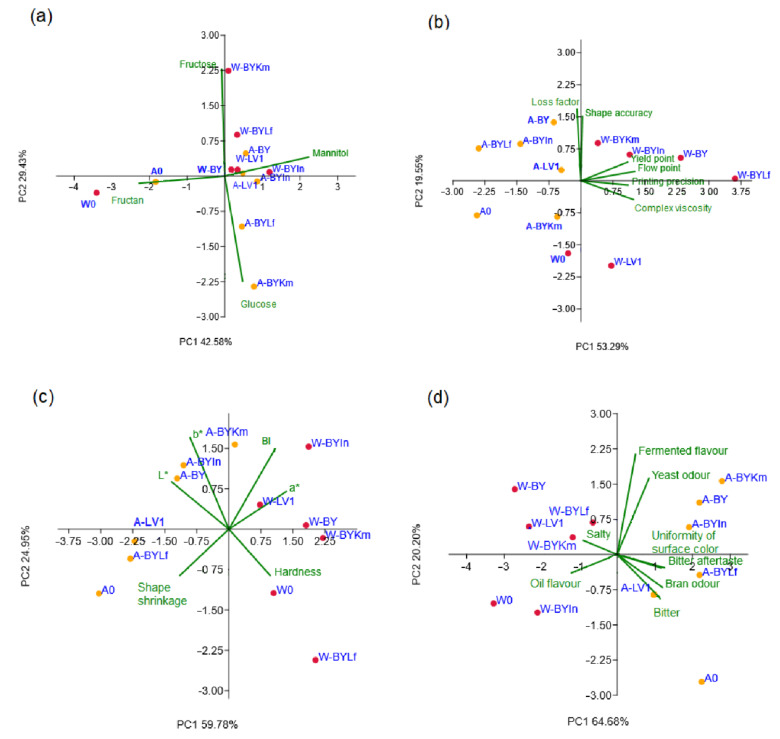
PCA bi-plots of investigated parameters: FODMAPs content (**a**), dough parameters (**b**), physical properties of snacks (**c**), and sensory attributes of snacks (**d**). W, wheat bran; A, amaranth bran; 0, control sample with unfermented bran; BY, bakery yeast; Lf, *Limosilactobacillus fermentum*; Km, *Kluyveromyces marxianus*; In, inulinase.

Results of the descriptive sensory analysis showed that there was a statistically significant difference between the samples in all evaluated attributes. A significant (*p* ≤ 0.02) dependence on the interaction between bran and bioprocessing agent type was observed for the bitter aftertaste, saltiness, and bran odour. Compared to wheat snacks, amaranth snacks, on average, had a more uniform colour, more pronounced bitter taste, yeast and bran odour, and less pronounced oil flavour (*p* < 0.01 for all attributes), which can be observed in Figure 4d. Although snacks with unprocessed WB had the least pronounced bitter aftertaste, the bioprocessing of WB with BY resulted in the least bitter snacks. As expected, the fermented flavour was significantly (*p* < 0.01) more expressed in both wheat and amaranth snacks after bran bioprocessing. Overall, bioprocessing attenuated the intensity of bran odour in amaranth snacks, whereby in wheat snacks, this was achieved only after treatments with LV1 and BY alone or combined with inulinase. ANOVA showed that the five-point hedonic scale did not detect any significant difference (*p* ≥ 0.09) in liking amongst the samples. The hedonic score of wheat snacks ranged from 2.7 to 3.8, and for amaranth snacks, it was between 2.6 and 3.3. This indicated a need for further improvement of the snack formulation to meet consumers’ expectations.

### 3.4. PCA

The results obtained using principal component analysis in the form of bi-plots correlating obtained snack products and analysing a set of variables are given in Figure 4a–d.

The negative correlation between fructans and mannitol content was obtained in bran samples (r = −0.689, *p* ≤ 0.05). The PCA of the presented data explained that the first two components accounted for 72.0% of the total variance in the four variables factor space (sugar contents). Considering the map of the PCA performed on the data, the contents of mannitol (contributing 47.7% of the total variance, based on correlations) exhibited positive scores according to the first principal component (PC1), whereas fructans content (50.0%) showed negative score values according to PC1 (Figure 4a). The positive contribution to the second principal component (PC2) calculation was observed for fructose content (49.9%), while negative scores on PC2 calculation were observed for glucose content (48.3%). Figure 4a explicitly show the abundance of fructans in unprocessed samples W0 and A0, whereas bioprocessed samples were characterised either by fructose, glucose, or mannitol presence, as discussed in Section 3.1. Regarding FODMAPs, the map of PCA analysis of samples showed that the second principal component described the differentiation among WB and AB, while the first principal component described the variations in the bioprocessing between samples.

According to Figure 4b, loss factor and shape accuracy were expressed in the dough with added amaranth bran bioprocessed with BY or LV1 and dough with wheat bran bioprocessed with yeasts co-culture, while other 3D printing parameters were being expressed in fermented snack samples made of wheat bran. The complex viscosity was positively correlated with yield point (r = −0.676; *p* ≤ 0.05), flow point (r = −0.909; *p* ≤ 0.01), and printing precision (r = −0.729; *p* ≤ 0.01). The PCA of the presented data explained that the first two components accounted for 72.84% of the total variance in the six variables factor space (3D printing parameters). The complex viscosity (contributing 27.4% of the total variance, based on correlations), yield point (21.7%), flow point (28.6%), and printing precision (22.1%) showed positive scores according to PC1 (Figure 4b). The positive contribution to PC2 calculation was observed for loss factor (50.8%) and shape accuracy (41.0%).

Figure 4c show that hardness was most expressed in WB-containing snacks either unfermented or fermented with BY and *L. fermentum*, while shrinkage characterised snacks containing AB unfermented or fermented with BY. The hardness was negatively correlated to diameter (r = −0.699; *p* ≤ 0.05). The browning index was positively correlated with a* colour coordinate (r = 0.688; *p* ≤ 0.05). The PCA of the presented data (Figure 4c) explained that the first two components accounted for 84.73% of the total variance in the six variables factor space (baked snack parameters). The shape shrinkage (which contributed 8.4% of the total variance, based on correlations), L* colour coordinate (15.5%), and b* colour coordinate (8.0%) showed positive scores according to PC1 (Figure 4c), whereas a negative contribution to PC1 calculation was obtained by BI (10.0%), a* colour coordinate (17.9%), and hardness (19.8%). A positive contribution to PC2 calculation was observed for BI (11.7%), while a negative influence on PC2 calculation was obtained for shape shrinkage (21.7%).

As shown in Figure 4d, fermented flavour and yeast odour were expressed in snacks with WB bioprocessed with BY and *L. fermentum* and AB bioprocessed with BY alone or in combination with *K. marxianus* or inulinase. Bitter taste and aftertaste, bran odour, and uniformity of surface colour were most expressed in amaranth snacks with unprocessed and LV1 or BY and *L. fermentum* co-culture-bioprocessed bran. Salty taste and oil flavour were characteristics of WB snack samples. The PCA of the presented data explained that the first two components accounted for 84.88% of the total variance in the eight variables factor space (sensory properties). The yeast odour (contributing 7.5% of the total variance, based on correlations), bran odour (14.5%), bitter taste (14.5%), and bitter aftertaste (16.9%) showed positive scores according to PC1, while a negative contribution to PC1 calculation was obtained by the uniformity of surface colour (13.7%), salty taste (9.1%), and oil flavour (14.5%). A positive contribution to PC2 calculation was observed for fermented flavour (50.7%) and yeast odour (29.4%), while a negative impact on PC2 calculation was observed for bitter taste (9.9%).

On average, wheat snacks were harder, darker, redder, saltier, had lower shrinkage and surface colour uniformity, and had more pronounced oil flavour while less pronounced bran flavour, bitter taste, and aftertaste than amaranth snacks.

## 4. Conclusions

In this study, we report for the first time the bioprocessing of wheat and amaranth bran with yeast, lactic acid bacteria, and inulinase, aimed at removing fructans, as well as improving the quality of 3D-printed snacks. Compared to amaranth bran, wheat bran contains a higher level of dietary fibre but also fructans. Bioprocessing lowers the fructans content in both brans, whereby the fructose released and the mannitol formed need to be followed. In addition, bioprocessing of the bran improves overall dough rheology, the precision of 3D printing, minimises shrinkage in baking, and contributes to the desired texture of the snacks. Bakery yeast successfully fermented wheat bran, assuring snack sensory and 3D printing quality. Overall, bioprocessed wheat bran at level 7% in the formulation could be used to produce low-FODMAPs snacks and labelled as a source of fibre. Amaranth bran has a further potential to enrich gluten-free snacks, particularly after bioprocessing with *K. marxianus*. Three-dimensional printing enables the fabrication of satisfactory snack products using milling by-products as an enriching ingredient intended for sensitive individuals and IBS patients. Future studies should investigate the shelf-life and cost efficiency of 3D-printed snacks with added bran to advance the sustainability of the food industry.

## Figures and Tables

**Table 1:** FODMAPs and glucose content in wheat and amaranth bran (% dry matter) and estimated total FODMAPs content in a serving of baked snacks (g/30 g).

Bioprocessing	Fructans	Fructose	Glucose	Mannitol	Total FODMAPs
Wheat bran					
None	2.64 ± 0.04 ^a^	0.14 ± 0.05 ^c^	0.47 ± 0.00 ^efgh^	n.d.	0.12
LV1	0.18 ± 0.01 ^de^	0.06 ± 0.00 ^c^	0.03 ± 0.00 ^i^	0.47 ± 0.01 ^fgh^	0.06
BY	0.96 ± 0.02 ^b^	0.09 ± 0.03 ^c^	0.24 ± 0.02 ^ghi^	0.65 ± 0.03 ^bc^	0.08
BY + *L. fermentum*	0.33 ± 0.02 ^c^	0.51 ± 0.06 ^b^	0.09 ± 0.04 ^i^	0.52 ± 0.01 ^def^	0.08
BY + *K. marxianus*	0.30 ± 0.01 ^c^	1.47 ± 0.16 ^a^	0.52 ± 0.08 ^cdef^	0.44 ± 0.01 ^gh^	0.12
BY + inulinase	0.19 ± 0.02 ^de^	n.d.	0.25 ± 0.02 ^fghi^	0.74 ± 0.02 ^a^	0.06
Amaranth bran					
None	0.96 ± 0.00 ^b^	0.13 ± 0.02 ^c^	0.17 ± 0.00 ^i^	n.d.	0.07
LV1	0.11 ± 0.01 ^ef^	n.d.	0.02 ± 0.00 ^i^	0.49 ± 0.02 ^efg^	0.05
BY	0.05 ± 0.00 ^f^	0.30 ± 0.02 ^c^	0.20 ± 0.01 ^hi^	0.50 ± 0.01 ^efg^	0.05
BY + *L. fermentum*	0.11 ± 0.00 ^ef^	n.d.	2.34 ± 0.10 ^b^	0.41 ± 0.00 ^h^	0.05
BY + *K. marxianus*	0.19 ± 0.01 ^de^	n.d.	5.10 ± 0.09 ^a^	0.45 ± 0.01 ^fgh^	0.06
BY + inulinase	0.08 ± 0.00 ^f^	n.d.	0.05 ± 0.02 ^defg^	0.58 ± 0.02 ^c^	0.05

n.d. not detected. Different letters within the same column indicate significant differences (*p* < 0.05). BY, bakery yeast.

**Table 2:** Rheological properties and 3D printing precision of the dough.

Treatment	Complex Viscosity (Pa s)	Yield Stress (Pa)	Flow Point (Pa)	Printing Precision (%)
Wheat bran				
None	7893.2 ± 131.2 ^de^	18.9 ± 0.3 ^f^	254.8 ± 0.6 ^ef^	84.3 ± 2.6 ^defg^
LV1	8184.1 ± 185.0 ^cd^	25.0 ± 1.0 ^ef^	447.2 ± 0.8 ^c^	86.3 ± 3.1 ^cdef^
BY	10,466.2 ± 182.2 ^b^	34.0 ± 1.0 ^cde^	533.8 ± 0.5 ^b^	93.1 ± 2.8 ^a^
BY + *L. fermentum*	11,275.5 ± 27.5 ^a^	51.4 ± 1.0 ^a^	612.0 ± 1.6 ^a^	94.9 ± 2.4 ^a^
BY + *K. marxianus*	7182.2 ± 11.8 ^e^	27.0 ± 1.0 ^def^	467.4 ± 0.6 ^c^	84.0 ± 2.7 ^efg^
BY + inulinase	8632.3 ± 15.9 ^cd^	26.4 ± 0.7 ^def^	472.5 ± 8.9 ^c^	88.4 ± 4.0 ^b^
Amaranth bran				
None	2237.6 ± 24.1 ^i^	5.1 ± 0.7 ^g^	126.9 ± 1.0 ^g^	83.3 ± 2.6 ^fg^
LV1	4540.0 ± 139.5 ^f^	37.4 ± 3.2 ^bcd^	301.3 ± 4.4 ^de^	82.8 ± 2.7 ^fg^
BY	3326.3 ± 73.1 ^gh^	27.9 ± 4.7 ^def^	290.9 ± 2.2 ^de^	86.1 ± 2.6 ^cdef^
BY + *L. fermentum*	2931.7 ± 182.5 ^hi^	2.2 ± 0.4 ^g^	229.3 ± 2.4 ^f^	80.1 ± 1.2 ^g^
BY + *K. marxianus*	4879.6 ± 209.2 ^f^	7.3 ± 0.1 ^g^	290.9 ± 6.7 ^de^	90.8 ± 2.0 ^a^
BY + inulinase	2503.4 ± 125.3 ^i^	20.8 ± 3.1 ^f^	221.3 ± 2.5 ^f^	84.0 ± 3.4 ^efg^

Different letters within the same column indicate significant differences (*p* < 0.05). BY, bakery yeast.

**Table 3:** Physical properties of baked snacks.

Bioprocessing	Shape Shrinkage (%)	Lightness L*	Redness a*	Yellowness b*	BI	Hardness (N)
Wheat bran						
None	28.6 ± 4.1 ^de^	57.8 ± 2.0 ^cd^	5.8 ± 1.3 ^efg^	17.2 ± 0.4 ^c^	42.3 ± 3.6 ^ef^	12.0 ± 1.6 ^bcd^
LV1	22.6 ± 5.3 ^fgh^	60.3 ± 0.9 ^b^	6.1 ± 0.4 ^def^	18.8 ± 0.7 ^defg^	44.2 ± 1.5 ^cde^	11.1 ± 1.3 ^defg^
BY	22.1 ± 3.4 ^gh^	56.7 ± 1.0 ^de^	7.6 ± 0.2 ^bc^	17.0 ± 0.8 ^bc^	44.8 ± 1.2 ^cde^	10.2 ± 0.7 ^efgh^
BY + *L. fermentum*	22.2 ± 2.9 ^gh^	53.7 ± 1.8 ^f^	5.6 ± 1.0 ^fgh^	15.3 ± 1.1 ^a^	40.6 ± 1.7 ^fg^	12.3 ± 1.4 ^abcd^
BY + *K. marxianus*	12.3 ± 2.3 ^j^	55.0 ± 0.4 ^ef^	6.9 ± 0.3 ^cd^	16.4 ± 0.3 ^bc^	44.0 ± 0.7 ^cde^	10.1 ± 0.6 ^efgh^
BY + inulinase	20.8 ± 3.2 ^hi^	60.1 ± 0.3 ^b^	7.7 ± 0.1 ^ab^	20.0 ± 0.2 ^fghi^	48.2 ± 0.3 ^a^	11.7 ± 2.0 ^cdef^
Amaranth bran						
None	48.2 ± 4.9 ^a^	64.9 ± 0.8 ^a^	3.3 ± 0.2 ^i^	19.0 ± 0.6 ^efgh^	37.8 ± 1.1 ^h^	10.0 ± 1.4 ^fgh^
LV1	31.6 ± 3.6 ^bcd^	64.5 ± 0.5 ^a^	3.5 ± 0.2 ^i^	19.3 ± 0.3 ^fghi^	39.0 ± 1.0 ^gh^	9.7 ± 0.6 ^gh^
BY	29.2 ± 4.5 ^cde^	64.4 ± 1.5 ^a^	5.0 ± 0.3 ^h^	20.2 ± 0.4 ^i^	42.7 ± 1.0 ^def^	10.1 ± 0.9 ^fgh^
BY + *L. fermentum*	30.3 ± 4.8 ^cde^	63.8 ± 0.7 ^a^	3.4 ± 0.3 ^i^	18.6 ± 0.8 ^def^	37.8 ± 2.0 ^h^	9.1 ± 0.7 ^h^
BY + *K. marxianus*	15.1 ± 3.1 ^ij^	60.9 ± 0.8 ^b^	5.3 ± 0.2 ^gh^	20.0 ± 0.6 ^ghi^	45.1 ± 1.3 ^bcd^	9.6 ± 1.3 ^gh^
BY + inulinase	25.2 ± 2.5 ^efgh^	64.3 ± 2.0 ^a^	5.3 ± 0.3 ^gh^	20.1 ± 1.1 ^hi^	42.7 ± 1.5 ^def^	9.6 ± 1.0 ^gh^

Different letters within the same column indicate significant differences (*p* < 0.05). BY, bakery yeast; BI, browning index.

## Data Availability

The data used to support the findings of this study are included in the article.
